# Whole-Genome Sequencing and Annotation of Exiguobacterium sp. RIT 452, an Antibiotic-Producing Strain Isolated from a Pond Located on the Campus of the Rochester Institute of Technology

**DOI:** 10.1128/MRA.01341-18

**Published:** 2018-11-01

**Authors:** Anutthaman Parthasarathy, Narayan H. Wong, Nicole T. Cavanaugh, KayLee K. Steiner, Peter C. Wengert, Michael A. Savka, André O. Hudson

**Affiliations:** a Thomas H. Gosnell School of Life Sciences, Rochester Institute of Technology, Rochester, New York, USA; Queens College

## Abstract

Exiguobacterium sp. RIT 452 is of biotechnological importance given its potential for antibiotic production. Bactericidal activity was detected using spent medium extract in a disk diffusion assay against Escherichia coli. The genome consists of 3,246 protein-coding sequences, including a variety of gene clusters involved in the synthesis of antibacterial compounds.

## Abstract (precis)

Exiguobacterium sp. RIT 452 is of biotechnological importance given its potential for antibiotic production.

## ANNOUNCEMENT

Exiguobacterium is a genus of Gram-positive soil bacteria widely distributed in the environment, from tropical (1) to polar regions (2). A number of species have been sequenced and were shown to be extremophiles, such as hyperthermophiles, alkaliphiles, halophiles, and psychrophiles (2–4). Other members of this genus have potential applications in the bioremediation of pesticides and metals and other applications in the biotechnology industry (5–11). One strain was found to produce antimicrobial compounds (1). A strain was isolated from the guts of mealworms, and it was shown that the bacterium was able to degrade the plastic polymer polystyrene (12).

We embarked on a project to isolate and identify bacteria that are able to produce bactericidal compounds from a pond located on the campus of the Rochester Institute of Technology (RIT). The bacterium was isolated by directly plating 100 µl of pond water sample on tryptic soy agar and growing it at 30°C under aerobic conditions. The bacterium was initially identified using PCR amplification and nucleotide sequencing of the 16S rRNA gene variable (V3/V4) regions using the following primers: 5′-CCTACGGGNGGCWGCAG-3′ and 5′-GACTACHVGGGTATCTAATCC-3′.

Genomic DNA was isolated from a 5-ml culture grown in tryptic soy broth using the GenElute bacterial genomic DNA isolation kit (Sigma-Aldrich, USA) according to the manufacturer’s protocol. For whole-genome sequencing, the genomic DNA was quantified using a NanoDrop spectrophotometer, and the genomic DNA was processed using the Nextera XT (Illumina) library preparation kit for sequencing using the MiSeq Illumina platform at the Rochester Institute of Technology Genomics Facility. Adapter trimming was done using the MiSeq Reporter software using the default parameters (sequences with >90% sequence identity to adapter sequences were trimmed). The trimmed reads were subsequently assembled *de novo* with Unicycler version 0.3.0b (13). An assembly of 1.76 million Illumina paired-end reads generated 27 contigs with a total length of 3,217,892 bp, an *N*_50_ value of 693,695 bp, and a GC content of 47.86%. The National Center for Biotechnology Information (NCBI) Prokaryotic Genome Annotation Pipeline (PGAP) predicted 3,246 protein-coding sequences, 5 rRNAs, and 67 tRNAs (14, 15).

**FIG 1:**
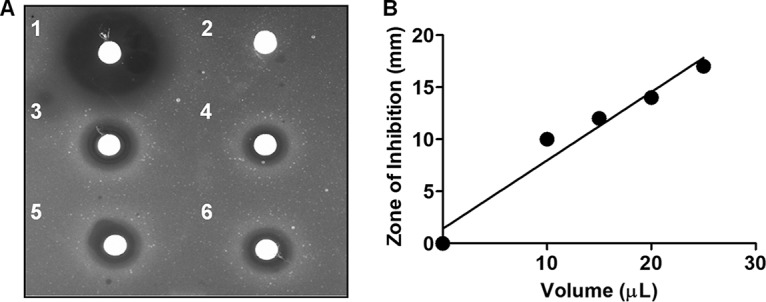
(A) Disk diffusion assay using ethyl acetate extract of spent medium of Exiguobacterium sp. RIT 452 against Escherichia coli ATCC 25922. (1) Tetracycline, 20 µl (10 mg/ml); (2) methanol, 20 µl; and (3, 4, 5, and 6) 25 µl, 10 µl, 20 µl, and 15 µl of RIT452 extract, respectively. (B) Diameter of the zone of inhibition (ZOI) showing the positive correlation between ZOI and volume.

A scan of the genome using the antibiotics and secondary metabolite analysis shell (antiSMASH4.0) webserver showed evidence that the bacterium has 27 gene clusters potentially encoding pathways for the synthesis of secondary metabolites, including carotenoids, other terpenes, and possibly antibiotics (16). A summary of the results highlights 4 of the 27 clusters (Table 1). With regard to the production of bactericidal compounds, the antiSMASH *in silico* analysis was corroborated by a disk diffusion inhibitory assay against Escherichia coli ATCC 25922 using ethyl acetate extract from Exiguobacterium sp. RIT 452 (Fig. 1).

**TABLE 1:** Summary of antiSMASH results for Exiguobacterium sp. RIT 452

Cluster no.	Predicted biosynthetic metabolite	Coordinates within the genome	% similarity to known cluster
1	Terpene	69899–90726	33 (with carotenoid_biosynthetic_gene_cluster)
4	Siderophore	505835–519165	
9	Putative antibiotic	897206–908755	26 (with lugdunin_biosynthetic_gene_cluster)
20	Terpene	160337–181161	

### Data availability.

This whole-genome project for Exiguobacterium sp. RIT 452 has been deposited in GenBank under accession number QXJB00000000. The version described in this paper is the first version, QXJB01000000 (BioProject number PRJNA489292; BioSample number SAMN09954399).
